# Corticosteroids for Managing TRK Inhibitor Withdrawal Pain: A Report on Two Cases

**DOI:** 10.3390/curroncol33020075

**Published:** 2026-01-27

**Authors:** Nicolas Marcoux, Louis-Philippe Grenier

**Affiliations:** 1 Medical Oncology, Centre Hospitalier Universitaire de Québec Université Laval, Québec, QC G1V 0A6, Canada; 2 Département de Pharmacie, Centre Intégré de Cancérologie (CIC), Centre Hospitalier Universitaire de Québec Université Laval, Québec, QC G1V 0A6, Canada

**Keywords:** NTRK inhibitor, larotrectinib, entrectinib, withdrawal symptoms, glucocorticoids

## Abstract

**Background:** Neurotrophin receptor tyrosine kinase (NTRK) fusions are potent oncogenic mutations. Inhibitors such as larotrectinib, entrectinib and repotrectinib are used when cancer cells harbor NTRK1, NTRK2 or NTRK3 fusion. Signal disruption between nerve growth factor (NGF) and its target is thought to impact nociception. Withdrawal pain is reported with larotrectinib and entrectinib. **Case presentation:** Two male patients aged 37 and 41 years old and treated with, respectively, repotrectinib and larotrectinib for NTRK fusion-positive solid tumors experienced debilitating pain after abrupt cessation of their targeted therapy. Short courses of prednisone for the former and dexamethasone for the latter were initiated after failure of standard analgesia. Both patients improved within 24 h and the pain did not recur after steroids were weaned off. They had improvements in their functional status without unexpected toxicity. **Conclusions and relevance:** For patients experiencing TRK inhibitor withdrawal pain, especially when tapering down the inhibitor is not an available strategy, a short course of corticosteroids can provide lasting relief. These cases emphasize the importance of better understanding the mechanism underlying the relationship between NRTK, NGF and nociception.

## Simple Summary

NTRK gene fusions are oncogenic drivers. Targeted TRK inhibitors such as larotrectinib, entrectinib and repotrectinib are effective therapies for tumors harboring these fusions. Abrupt cessation of TRK inhibitors can trigger withdrawal pain. A short course of corticosteroids (prednisone or dexamethasone) may provide rapid and lasting relief from TRK inhibitor withdrawal pain, highlighting a potential therapeutic approach when tapering is not feasible.

## 1. Introduction

Much has been learned since the approval of the first tyrosine kinase inhibitor (TKI) by the FDA in 2001. This was preceded by the recognition of the *BCR-ABL* fusion as responsible for nearly all the cases of chronic myeloid leukemia and for a significant subset of acute lymphoid leukemias, followed by the development of an inhibitory compound, imatinib [1]. In contrast to the disease-specific nature of this mutation, some oncogenic fusions can be involved in a wide range of cancers. Neurotrophin receptor tyrosine kinase (*NTRK1*, *NTRK2* or *NTRK3*) fusions encoding for tropomyosin receptor kinase TRKA, TRKB and TRKC [2] are great examples and are found in a variety of cancers. Larotrectinib has shown efficacy in a basket study involving adults and children with solid tumors harboring *NTRK* fusion-positive cancers [3]. Second-generation TKIs such as repotrectinib have also demonstrated activity, opening the door to treatment sequencing with targeted agents [4].

TRK receptors are involved in neuronal development, survival and function. On-target adverse events are predictable, as the impact of targeting TRKA, TRKB or TRKC has been described [2]. Paresthesia, weight gain as a result of hyperphagia and dizziness, all directly related to the inhibition of TRK receptors, are manageable by dose reduction [2,5]. The absence of nerve growth factor (NGF), the ligand for TRKA, during fetal development leads to insensitivity to pain [6]. It was also described that genetic mutations in *NTRK* were responsible for congenital insensitivity to pain, along with anhidrosis [7]. Animal models carrying a disrupted TRK/NGF receptor gene have severe neuropathies resulting from impaired central and peripheral nervous system development [8]. On the other hand, in order to manage intractable pain, some studies were conducted to suppress NGF/TRKA signaling [6]. These findings underline the importance of TRK activity in nociception, which is likely related to the development of severe pain upon TKI discontinuation [2,5,9].

Up to 34% of patients will report withdrawal pain after temporary or permanent discontinuation of the drug. Full-body aches, muscle pain, allodynia and headaches are listed as withdrawal symptoms. Slowly tapering the inhibitor seems partially effective, and gabapentin is reported to have limited impact. While the median onset of pain is 2 days (1–6 days), the median duration of withdrawal pain is 14 days (10–26) days for those with permanent discontinuation [9].

After obtaining the written consent of the patients or their relatives, we retrospectively analyzed the patients’ electronic medical records, and for one patient, we were able to obtain precision regarding the timeline by family members. We hereby report the details of what we believe represent withdrawal symptoms following TKI cessation and our novel pain management strategy.

## 2. Case Presentation

### 2.1. Case 1

We first report the case of a 37-year-old male. He had no drug allergy and he had a history of chronic osteitis, well managed with suppressive doxycycline therapy. Notable medication included a stable dose of pregabalin 125 twice daily, acetaminophen 650 mg up to three times a day and occasional ibuprofen. He was a non-smoker and used occasional alcohol. His body mass index was 40 kg^2^.

He was diagnosed 3 years earlier with a high-grade 13 cm thoracic undifferentiated spindle-cell sarcoma. It was first addressed with neoadjuvant 50 Gy radiotherapy, and two surgeries were needed to attain negative margins. He presented with lung and bone metastatic disease only four months after the second surgery. Shortly after a single doxorubicin cycle, next-generation sequencing (NGS) analysis on tumor tissue revealed a NTRK fusion (Table 1). Larotrectinib 100 mg BID was initiated, and first imaging revealed a near-complete response after 3 months. He then had stable disease for 14 months, requiring stereotaxic radiotherapy for lung oligoprogression at month 14. After subsequent progression, he enrolled in a clinical trial evaluating activity of repotrectinib in *NTRK*-mutated cancers and had an excellent response. He developed pleural and central nervous system disease progression after 9 months on trial. Five weeks prior, during a routine follow up visit, he reported an excellent performance status, with an Eastern Cooperative Oncology Group (ECOG) score 0/4.

Following disease progression, protocol required immediate cessation of repotrectinib. He complained of severe musculoskeletal pain within 1 week after drug discontinuation, despite ibuprofen, pregabalin and acetaminophen. He described muscle aches and “not recalling being in such a bad shape”. He also mentioned not being able to walk his daughter to school. Details on blood tests performed at that time are available in Table 1. Because of a severe impact on daily living activities and a declining performance status of ECOG = 1, prednisone 20 mg daily for 5 days was initiated, followed by prednisone 10 mg daily for 5 more days. He could return to activities of daily living as early as 24 h after the first dose. There was no recurrence of the muscle pain at follow-up after steroids cessation.

Four weeks after, during an outpatient palliative care evaluation that took place before initiation of the subsequent line of systemic therapy, the patient’s left thoracic and pleural discomfort was rated to 3/10 on the D rating scale (NRS), used in cancer pain evaluation [10]. There still was no mention of the musculoskeletal pain symptoms reported earlier.

### 2.2. Case 2

Our second case is a 41-year-old male who never smoked. His medical history was negative, except for an attention deficit/hyperactivity disorder. His medication included nasal ciclesonide, citalopram and lisdexamphetamine. He first presented with painful skin lesions and thoracic pain. He would later be diagnosed with metastatic large-cell neuroendocrine carcinoma of the lung. Imaging revealed extensive disease that had spread to brain, bones, liver, kidneys and soft tissue. A first dose of carboplatin–etoposide was administered before the availability of mutational status. Tumor analysis was positive for *NTRK3* fusion (Table 1) and larotrectinib was initiated. Disease control was suboptimal, with a mixed response that lasted no more than 6 months. At time of clear disease progression, larotrectinib was stopped with plans to restart cytotoxic chemotherapy.

Around 12 h after his last dose of larotrectinib, he reported generalized muscle pain, rated 5/10 on the NRS. Interestingly, he had previously described this type of pain during active treatment when he delayed larotrectinib doses for more than 12 h. During the next 2 days, the pain increased up to 8/10, despite around-the-clock morphine 5 mg orally every 4 h. He was using a wheelchair when he showed up to the outpatient clinic on day 3, suffering from muscle weakness and severe headache.

A carboplatin–etoposide protocol was initiated with dexamethasone 12 mg IV as premedication, and the headache resolved no more than 30 min later. The pain decreased to 3/10 and he was even more comfortable after a single dose of subcutaneous morphine 2.5 mg. This dexamethasone dose on day 1, followed by oral dexamethasone 4 mg twice daily for five doses, completely alleviated the muscle pain, which did not relapse after cessation.

## 3. Discussion

Larotrectinib, entrectinib and, more recently, repotrectinib were granted FDA approval for adult and pediatric solid tumors positive for *NTRK* fusion. The approach of molecular testing and targeted therapy demonstrated promising results in phase I-II studies [3,11,12]. The drugs are well tolerated, but there have been reports of withdrawal symptoms with larotrectinib and entrectinib. To our knowledge, this is the first report of repotrectinib withdrawal pain, which supports the hypothesis of a class effect resulting from an on-target mechanism. The TRK signaling pathway has been reported to contribute to heat and mechanical hypersensitivity and to persistence of pain in animal models [13]. During larotrectinib, entrectinib and repotrectinib treatment, on-target TRKB inhibition leads to impaired nociception and is thought to be responsible for withdrawal pain following interruption of therapy [9]. Increased nerve growth factor (NGF) signaling following TRK inhibitor discontinuation seems the most likely explanation, as it has been described that NGF and TRK regulate the nervous system and pain modulation [14]. We hypothesize that abrupt restoration of NGF-TRK signaling triggers compensatory overstimulation, causing pain, which can be stopped when reintroducing the NTRK inhibitor.

Tyrosine kinase inhibitor withdrawal symptoms were first reported after the trials for BCR-ABL inhibitor discontinuation in 2014 [15]. A substantial number of patients reported musculoskeletal pain that begins or worsens weeks after stopping imatinib therapy. The symptoms developed through months 1 (20%) to months 2–3 (73%) and were reported more frequently in females (58%) [16]. Acetaminophen and non-steroidal anti-inflammatories were used for mild symptoms; corticosteroids were given and tapered for patients unable to withstand the pain in everyday activities [15].

The activity of drugs frequently used for neuropathic pain, such as gabapentin, was previously described as very limited, and slowly tapering down the inhibitor is so far the only effective recommendation [14]. Although TRK inhibitor reintroduction was the most effective intervention, it is not always appropriate in settings of disease progression, clinical trials or severe toxicity requiring permanent drug interruption [9]. Chin et al. reported 5 out of 21 patients experiencing pain after discontinuation of entrectinib or larotrectinib [14]. As observed in our cases, the onset of pain ranged from a few hours to up to 3 days, and patients reported muscle aches, joint pain and weakness. Tramadol was reported to be partially effective, whereas anti-inflammatory drugs and acetaminophen were not.

Differential diagnosis includes cancer-related pain, as both patients had progressive disease at the time of initial symptoms. However, the diffuse nature of the muscle aches is discordant with the known sites of metastatic disease. Patient 1 had a period of more than a week between steroid cessation and initiation of subsequent systemic therapy, during which purely cancer-related pain would have been expected to reappear. The same conclusion would apply to patient 2, who had disease progression as best response to subsequent-line therapy, but without recurrence of pain. Contributing biochemical imbalances are unlikely in both cases. Viral infections should be taken in account, as both our cases occurred during peak viral seasons in Canada. While this remains possible in case 1 despite the absence of other upper respiratory tract symptoms, the precise timing and prior history of similar symptoms developing after brief interruptions in therapy for patient 2 strongly support a causal relationship. After ruling out tumor-related pain [17] and biochemical imbalance, we believe that treatment cessation remains the most probable explanation for the pain.

Management with a short course of corticosteroids, either prednisone or dexamethasone, was effective in alleviating severe pain for the two patients above. Reduction in the intensity of muscle pain and headache was fast and lasted after steroid cessation. While our experience is limited to the two patients above, it supports an inexpensive and safe approach for patients experiencing withdrawal symptoms. The mechanism by which corticosteroids might impact nociception, thought to be mediated by NGF/TRK dysregulation, remains uncertain, but preclinical work supports complex interactions between steroids and NGF expression [18].

Animal and human models have shown that corticosteroids have a variable effect on NGF expression depending on the tissue type and cell population. The interaction between glucocorticoids and the expression of NGF remains complex and depends on the studied tissues [19]. Glucocorticoid hormones have a negative regulation on NGF synthesis by repressing NGF gene transcription after sciatic transection in rats [20]. Based on the available body of literature, the impact of prednisone or dexamethasone on NTRK inhibitor withdrawal pain could by the result of a direct inhibitory effect on NGF expression.

## 4. Conclusions

These cases highlight the clinical importance of withdrawal symptoms following larotrectinib and repotrectinib and suggests a short course of corticosteroids as a strategy to manage the pain, especially when drug tapering is not available or feasible. More studies are needed to better understand the physiology of NTRK withdrawal pain and to explore the effect of corticosteroids used in this setting.

## Figures and Tables

**Table 1:** Summary of both clinical cases.

	Case 1	Case 2
Age	37 years	41 years
Cancer	Undifferentiated spindle-cell sarcoma	Large-cell neuroendocrine carcinoma
Specific mutation	NTRK1-TPM3 fusion	NTRK3-ETV6 fusion
Laboratory findings	Leucocytes 14.1 × 10^9^/L	Leucocytes 5 × 10^9^/L (4.2–10.5)
	Platelets 411 × 10^9^/L	Platelets 193 × 10^9^/L (150–400)
	Creatinine 77 μmol/L	Creatinine 87 umol/L (55–105)
	Bilirubin 7 μmol/L	Bilirubin 9 umol/L (0–17)
	TSH ^1^ 1.95 mUI/L (0.34–4.82)	TSH 0.64 (0.34–4.82)
		Cortisol 444 nmol/L ^2^
Targeted therapy	Repotrectinib	Larotrectinib
Duration of therapy before cessation	9 months	6 months
Elapsed time before onset of withdrawal symptoms	Within 1 week	12 h
Symptoms	Muscle pain	Musculoskeletal pain, weakness, headache
Corticosteroid	Prednisone 20 mg daily for 5 days	Dexamethasone 12 mg once
	Prednisone 10 mg daily for 5 days	Dexamethasone 4 mg twice a day for 5 doses
Time before improvement	24 h	<24 h
Recurrence of withdrawal symptoms	No	No

^1^ Thyroid-stimulating hormone. ^2^ Reference values 6 to 8 h AM 170–730 nmol/L. Values in parenthesis are the reference values for local laboratory.

## Data Availability

The original contributions presented in this study are included in the article. Further inquiries can be directed to the corresponding author.

## Abbreviations

The following abbreviations are used in this manuscript:

NGF	Nerve growth factor
NGS	Next-generation sequencing
NTRK	Neurotrophin receptor tyrosine kinase
TKI	Tyrosine kinase inhibitor
TRK	Tropomyosin receptor kinase
